# An improved ileal conduit surgery for bladder cancer with fewer complications

**DOI:** 10.1186/s40880-019-0366-8

**Published:** 2019-04-18

**Authors:** Zhiyong Li, Zhuowei Liu, Kai Yao, Zike Qin, Hui Han, Yonghong Li, Pei Dong, Yunlin Ye, Yanjun Wang, Zhiming Wu, Zhiling Zhang, Fangjian Zhou

**Affiliations:** Department of Urology, State Key Laboratory of Oncology in South China, Collaborative Innovation Center for Cancer Medicine, Sun Yat-sen University Cancer Center, No. 651 Dongfeng Road East, Guangzhou, 510060 Guangdong P. R. China

**Keywords:** Bladder cancer, Cystectomy, Urinary diversion, Ileal conduit, Complication, Surgical technique

## Abstract

**Background:**

Radical cystectomy and urinary diversion remains the standard surgical treatment for patients with muscle-invasive or high-risk or recurrent non-muscle-invasive bladder cancer. Although this approach prolongs patient survival remarkably, there are postoperative complications associated with urinary diversion. This study aimed to assess the efficacy of modified ileal conduit surgery for reducing early and late stoma- and ureteroileal anastomosis-related complications, as compared with conventional ileal conduit urinary diversion.

**Methods:**

We retrospectively evaluated the clinical data of bladder cancer patients treated with radical cystectomy and ileal conduit urinary diversion at Sun Yat-sen University Cancer Center between January 1, 2000 and June 30, 2016. Ileal conduit was created by the conventional or a modified technique. The clinicopathologic features of the conventional and the modified ileal conduit groups were compared using the *t* test and the Chi square test. Multivariable logistic regression analysis and multivariable Cox regression analysis were performed to determine the odds of developing stoma- and ureteroileal anastomosis-related complications in the two groups.

**Results:**

145 and 100 patients underwent the modified and conventional ileal conduit surgery, respectively. The two groups were comparable with regard to clinicopathologic features. The rate of stoma-related complications was significantly lower in the modified ileal conduit group than in the conventional ileal conduit group (0.7% vs. 17.0%, *P* < 0.001). No late stoma-related complications were seen in the modified ileal conduit group, but were seen in 13 (13.0%) patients in the conventional ileal conduit group. The rate of ureteroileal anastomosis-related complications was significantly lower in the modified ileal conduit group than in the conventional ileal conduit group (4.8% vs. 15.0%, *P* = 0.001). In multivariable analyses, the modified ileal conduit group was significantly less likely to develop stoma- (odds ratio [OR] = 0.024, 95% confidence interval [CI] 0.003–0.235; *P* = 0.001) or ureteroileal anastomosis-related complications (OR = 0.141, 95% CI 0.042–0.476; *P* = 0.002) than the conventional ileal conduit group.

**Conclusions:**

Our modified surgical technique for ileal conduit urinary diversion may be effective for reducing early and late complications related to the stoma and the ureteroileal anastomosis. Prospective randomized clinical trials are needed to confirm our results.

## Background

Bladder cancer is the eleventh most commonly diagnosed cancer worldwide and the seventh most common cancer among men [1]. Approximately 25% of patients with bladder cancer present with tumor-invading muscle [2]. For muscle-invasive bladder cancer and high-risk and recurrent non-muscle-invasive bladder cancer, radical cystectomy and urinary diversion remains the standard treatment [3]. Urinary diversion following radical cystectomy can be of two types: incontinent and continent. Incontinent urinary diversion consists of ureterocutaneostomy and ileal conduit urinary diversion. Continent diversion includes creation of an orthotopic neobladder and continent cutaneous urinary diversion. Most institutions prefer the creation of ileal orthotopic neobladders and ileal conduits, especially ileal conduit diversion (60%), based on data statistics [4, 5]. For ureterocutaneostomy either one ureter—to which the other shorter one is attached end-to-side—is connected to the skin or both ureters are directly anastomosed to the skin. Because of the short diameter of the stenosis of the ureter is likely at the anastomois, resulting in a higher frequency of ascending urinary tract infection after ureterocutaneostomy than after ileal conduit diversion [6]. For continent cutaneous urinary diversion, an intestinal segment is used to create a low-pressure urinary reservoir, connected to an opening in the abdominal wall with a catheterizable continence mechanism. In a retrospective study of more than 800 patients who underwent continent cutaneous urinary diversion, stomal stenosis was seen in 23.5% and stone formation in the pouch was seen in 10% of patients [7]. For orthotopic neobladder, a pouch (the neobladder or new bladder) is created inside the abdomen using a segment of the small intestine. In two studies with 1054 and 1300 patients, long-term complications following orthotopic neobladder surgery included diurnal (8% and 10%) and nocturnal incontinence (20% and 30%), ureterointestinal stenosis (3% and 18%), metabolic disorders (33% and 7%), and vitamin B12 deficiency (0.2% and 0%) [8, 9].

In ileal conduit urinary diversion, a segment of the intestine directs urine through a stoma into an external collecting bag. The ileal conduit (Bricker) has been used for urinary diversion for more than half a century. Widely accepted to be a simple and safe type of urinary diversion, it remains a commonly used technique for urinary diversion after radical cystectomy for bladder cancer [3, 10–13]. However, many complications may occur after ileal conduit diversion, with the rate increasing with time after surgery [14]. Interestingly, nearly half of the complications are related to stoma and ureteroileal anastomosis [15, 16]. Among the complications, parastomal hernia is the most common. Female gender, low preoperative serum albumin level, high body mass index (BMI) or severe obesity, and prior laparotomy have been shown to be risk factors for parastomal hernia [17, 18]. However, the mechanisms by which these factors lead to stoma- or ureteroileal anastomosis-related complications have not yet been fully elucidated. Several modified surgical techniques have been used to prevent stoma- and ureteroileal anastomosis-related complications. Pagano et al. [19] reported a low rate of complications in 100 consecutive patients who were treated with a modified technique for ileal conduit urinary diversion, in which the ileal conduit was placed in its natural isoperistaltic anterior position and the ureters were anastomosed in anterior positions on their respective sides. Taneja et al. [20] described a new stoma-creating technique that could improve stomal protrusion, eversion, and symmetry. Gillitzer et al. [21] used a modified technique for extra-peritonealisation of the ileal conduit in 9 patients and reported no parastomal hernia over a limited follow-up period. McGrath et al. [22] have proposed that the rate of parastomal hernia could be decreased by passing the stoma through the rectus abdominis instead of the abdominal wall lateral to the rectus abdominis. However, we believe that passing the stoma through the rectus abdominis is impractical in Asian patients because of their relatively small body build. In fact, in our previous study [23] and the present study the stoma was, without exception, located lateral to the rectus abdominis in all patients.

We believe that the surgical technique is responsible for these complications, and therefore have devised a modified technique for creating the ileal conduit and the ureteroileal anastomosis that should help prevent these complications after surgery. Initial results showed that complications related to stoma and ureteroileal anastomosis were effectively prevented with the use of our modified technique [23]. The present retrospective study was aimed to compare the rates of early and late stoma- and ureteroileal anastomosis-related complications between patients undergoing conventional and modified ileal conduit surgery.

## Patients and methods

### Patient selection

Clinical data of bladder cancer patients undergoing conventional or modified ileal conduit surgery at Sun Yat-sen University Cancer Center between January 1, 2000 and June 30, 2016 were reviewed. Patients were eligible for inclusion if they had (1) muscle-invasive bladder cancer (T2-4aN0-xM0), high-risk and recurrent non-muscle-invasive tumors (Bacille Calmette-Guerin [BCG]-resistant Tis, T1G3), or extensive papillary disease that was not controlled with transurethral resection of bladder cancer and intravesical therapy alone and therefore had been advised radical cystectomy and ileal conduit urinary diversion; (2) Eastern Cooperative Oncology Group (ECOG) score of 0–1; (3) normal functions of all major organs (e.g., the liver, kidney, bone marrow, heart); and (4) completed ≥ 6 months of follow-up. Patients were excluded if they had (1) history of major surgery in the middle or lower abdomen; (2) severe obesity (BMI ≥ 40 kg/m^2^); or (3) history of immunodeficiency or severe central nervous system disease.

The selection of modified or conventional ileal conduit surgery was based on the surgeon’s experience and the patient’s preference. In the initial 3 years of the study period, only conventional ileal conduit was performed. However, with accumulation of experience, the modified ileal conduit was increasingly adopted and was performed more often than conventional ileal conduit since 2009.

Data related to gender, age, smoking status, BMI, adjuvant chemotherapy, neoadjuvant chemotherapy, American Society of Anesthesiology (ASA) score, Charlson comorbidity index (CCI), prior pelvic irradiation, preoperative hydronephrosis, pathologic TNM stage, and follow-up examination results were collected. This study was performed in accordance with institutional ethical guidelines, and signed informed consent was obtained from all patients.

### Surgical technique

Conventional ileal conduit surgery (including radical cystectomy, pelvic lymph node dissection, and conventional ileal conduit diversion) was performed according to the technique described previously [24]. After cystectomy and lymph node dissection, the left ureter was transposed to the right side through a retrosigmoidal tunnel. A 15- to 20-cm segment of the ileum was isolated 15 cm proximal to the ileocecal valve. After restoring bowel continuity, the ureters were anastomosed to the ileal conduit in an end-to-side fashion with ureteral stents for 14 days. Then the distal end of the ileal segment was pulled out through the abdominal wall directly and anastomosed to the skin in a nipple-to-stoma fashion.

The technique of the modified ileal conduit diversion has been described in detail in our previous report [23]. Please see the brief video of the surgical process available at https://pan.baidu.com/s/13jsVs-jBqUkB_9ADzH3uOw. The stoma was created intracorporeally using the distal segment of the ileum, which was then passed through an extraperitoneal tunnel and fixed at a previously prepared site on the abdominal wall. The spatulated ureters and the conduit were connected by end-to-side anastomosis with a 3-0 absorbable continuous lock-stitch suture after the stoma was fixed to the skin. The ureters were dissected free as much as possible to achieve a tension-free ureteroileal conduit anastomosis [25]. A ureteral stent was placed before anastomosis and was removed after anastomosis. A 24-Fr multiorifice catheter was placed in the ileal conduit and was removed only after full recovery of gastrointestinal peristalsis. The proximal end of the conduit was closed, and the peritoneum was sutured over the conduit and ureters to make the ileal conduit and the ureteroileal anastomosis completely extraperitoneal.

### Follow-up evaluation

Follow-up was planned according to our institutional protocol, with patients evaluated every 3 months for the first 2 years after surgery and every 6 months thereafter. Follow-up evaluations included physical examination, blood chemistry tests, and ultrasonography of the upper urinary tract. Intravenous pyelography (IVP) and computed tomography (CT) of the abdomen and pelvis were ordered if there was any evidence of upper urinary tract dilatation or tumor recurrence. Urine cultures were performed only when urinary infection was suspected. The last follow-up was in December 2016.

### Definition of complications

Only stoma- and ureteroileal anastomosis-related complications were analyzed in this study. Complications were defined as early complications, occurring within 3 months after surgery, or late complications, developing 3 months after surgery. The complications were graded according to the Clavien Grading System [26].

Stoma-related complications included parastomal hernia, stomal stenosis, stomal retraction, stomal prolapse, and stomal necrosis. Parastomal hernia was defined as protrusion of abdominal contents through the abdominal wall defect in the vicinity of the stoma, leading to a bulge at the base of stoma [27]. CT scan or magnetic resonance imaging of the abdomen was used to confirm the diagnosis of parastomal hernia. Stomal stenosis was defined as narrowing of the stoma at the skin or fascia, leading to impaired urinary drainage [28]. Stomal prolapse was defined as significant increase in stoma size or length after maturation [29]. Stomal retraction was diagnosed when the stoma appeared sunken or was depressed below the level of the abdominal wall [30]. Mild stomal ischemia, which was primarily due to operative tissue trauma or vasospasm, was diagnosed when there was mucosal sloughing or infarction. Stomal necrosis, which was due to inadequate collateral arterial circulation or ligation of arterial supply to the exteriorized segment of bowel, was diagnosed when there was a change in stoma viability or tissue death [31].

Ureteroileal anastomosis-related complications included urinary leakage from the ureteroileal anastomosis and obstruction at ureteroileal anastomotic site. Urinary leakage was diagnosed when the creatinine level of pelvic drainage was elevated and leakage from the proximal end of the conduit was excluded. Obstruction was diagnosed when ultrasonography or IVP showed dilatation of the whole upper urinary tract [32].

### Statistical analysis

Continuous variables were summarized as mean values and standard deviations (SD) for parametric distribution or median values and interquartile ranges (IQR) for nonparametric distribution. The *t* test or Mann–Whitney *U* test (continuous variables) and the Chi square test (categorical variables) were applied to assess differences between the two groups. Due to the high degree of similarity in the distribution of variables in the two groups, propensity score-matching analysis was not considered necessary. Multivariable logistic regression models and multivariable Cox regression models adjusted for different covariates (gender, age, smoking status, ASA, BMI, CCI, prior pelvic irradiation, preoperative hydronephrosis, year of surgery, adjuvant chemotherapy and pathologic TNM stage) were used for the prediction of risk of stoma- and ureteroileal anastomosis-related complications in the two groups. All *P* values were two-sided, with *P *< 0.05 indicating statistical significance. Statistical analysis was performed using SPSS 22.0 (IBM Corp., Armonk, NY, USA).

## Results

### Patient characteristics

Of the 326 patients considered for inclusion in the study, 245 met the eligibility criteria. Among these 245 patients, 100 underwent conventional ileal conduit surgery, and 145 underwent modified ileal conduit surgery. Table 1 lists the demographic and clinicopathologic characteristics of the 245 patients. Eleven patients underwent neoadjuvant chemotherapy. There was no significant difference between the two groups in baseline characteristics. Median follow-up for the 245 patients was 25.0 months (range, 6–180 months): 18.5 months (range, 6–180 months) for the conventional ileal conduit group versus 26.0 months (range, 6–156 months) for the modified ileal conduit group (*P *= 0.560).

**Table 1:** Demographic characteristics of 245 bladder cancer patients who underwent ileal conduit urinary diversion

Characteristic	Entire cohort	Conventional ileal conduit group	Modified ileal conduit group	*P* value
Total (cases)	245	100	145	
Age [years, median (range)]	62 (18–89)	62 (30–89)	62 (18–88)	0.733
Gender [cases (%)]				0.684
Male	199 (81.2)	80 (80.0)	119 (82.1)	
Female	46 (18.8)	20 (20.0)	26 (17.9)	
Smoking status [cases (%)]				0.762
Yes	127 (51.8)	53 (53.0)	74 (51.0)	
No	118 (48.2)	47 (47.0)	71 (49.0)	
Adjuvant chemotherapy [cases (%)]				0.653
Yes	131 (53.5)	61 (61.0)	70 (48.3)	
No	114 (46.5)	39 (39.0)	75 (51.7)	
Neoadjuvant chemotherapy [cases (%)]				0.759
Yes	11 (4.5)	4 (4.0)	7 (4.8)	
No	234 (95.5)	96 (96.0)	138 (95.2)	
BMI [kg/m^2^, mean ± SD]	21.8 ± 3.2	22.2 ± 2.8	21.3 ± 3.6	0.834
CCI [cases (%)]				0.902
≤ 2	175 (71.4)	71 (71.0)	104 (71.7)	
> 2	70 (28.6)	29 (29.0)	41 (28.3)	
ASA score [cases (%)]				0.613
≤ 2	183 (74.7)	73 (73.0)	110 (75.9)	
> 2	62 (25.3)	27 (27.0)	35 (24.1)	
Prior pelvic irradiation [cases (%)]				0.072
Yes	5 (2.0)	4 (4.0)	1 (0.7)	
No	240 (98.0)	96 (96.0)	144 (99.3)	
Preoperative hydronephrosis [cases (%)]				0.532
Yes	79 (32.2)	30 (30.0)	49 (33.8)	
No	166 (67.8)	70 (70.0)	96 (66.2)	
Pathologic T stage [cases (%)]				0.129
1	45 (18.4)	25 (25.0)	20 (13.8)	
2	57 (23.3)	21 (21.0)	36 (24.8)	
3	81 (33.1)	33 (33.0)	48 (33.1)	
4	62 (25.3)	21 (21.0)	41 (28.3)	
Pathologic N stage [cases (%)]				0.797
0	152 (62.0)	63 (63.0)	89 (61.4)	
≥ 1	93 (38.0)	37 (37.0)	56 (38.6)	
Year of surgery				0.052
Before/in 2009	42 (17.1)	11 (11.0)	31 (21.4)	
After 2009	203 (82.9)	89 (89.0)	114 (78.6)	

*BMI* Body mass index, *SD* standard deviations, *CCI* Charlson comorbidity index, *ASA* American Society of Anesthesiology

As our modified technique was mainly used for preventing stoma- and ureteroileal anastomosis-related complications, we did not study other complications in detail. However, there was no significant difference between the modified and conventional group in the rates of other major complications, such as bowel obstruction, impaired renal function, and urinary tract infection (Table 2).

**Table 2:** Major complications related to bowel obstruction, renal function and urinary tract infection in bladder cancer patients after ileal conduit surgery

Complication	Conventional ileal conduit group [cases (%)]	Modified ileal conduit group [cases (%)]	*P* value
Bowel obstruction	15 (15.0)	19 (13.1)	0.673
Impaired renal function	14 (14.0)	20 (13.8)	0.963
Urinary tract infection	5 (5.0)	10 (6.9)	0.543

### Early complications related to the stoma and ureteroileal anastomosis

A total of 21 early complications related to the stoma and ureteroileal anastomosis were seen in 19 (7.8%) of the 245 patients (Table 3).

**Table 3:** Early complications related to stoma and ureteroileal anastomosis in bladder cancer patients after ileal conduit surgery

Complication	Conventional ileal conduit group (cases)	Modified ileal conduit group (cases)
Total	12	9
Stomal necrosis		
CDC I	1	0
CDC IIIb	3	1^a^
Ureteroileal anastomosis leakage		
CDC I	2	1
CDC IIIa	1	1
CDC IIIb	0	2
Ureteroileal anastomosis obstruction		
CDC I	4	1
CDC IIIa	1^b^	2^c^
CDC IIIb	0	1

*CDC* Clavien-Dindo classification

^a^The patient presented with mild stomal ischemia

^b^One patient had concurrent CDC IIIa ureteroileal anastomosis leakage

^c^One patient had concurrent CDC IIIa ureteroileal anastomosis leakage

In the conventional ileal conduit group, early complications occurred in 11 (11.0%) of the 100 patients; 4 patients had stomal necrosis alone, 2 had ureteroileal anastomosis leakage alone, 4 had ureteroileal anastomosis obstruction alone, and 1 had both ureteroileal leakage and ureteroileal anastomosis obstruction. Of the 4 patients with stomal necrosis, 3 were successfully treated by removal of the old ileal conduit and creation of a new ileal conduit; the remaining 1 patient, who had mild stomal necrosis, recovered with conservative treatment. The 3 patients with ureteroileal anastomosis leakage (unilateral in 2 patients and bilateral in 1 patient) were treated conservatively by maintaining urinary drainage; 2 recovered without upper urinary tract dilatation, 1 developed obstruction at the ureteroileal anastomosis and needed endoscopic incision and ureteral stenting. The 5 patients with mild ureteroileal anastomosis obstruction (bilateral in 4 patients and unilateral in 1 patient) and hydronephrosis were managed with close surveillance; the hydronephrosis gradually disappeared in all patients within 3 months after surgery, without any late sequelae.

In the modified ileal conduit group, early complications were seen in 8 (5.5%) of the 145 patients. One patient developed mild stomal ischemia 5 days after surgery. Emergent exploratory laparotomy revealed that the stomal ischemia was caused by severe ileus. The patient recovered after the ileus was relieved. Urine leakage from the ureteroileal anastomosis was seen in 4 patients (unilateral in 2 patients and bilateral in the other 2 patients), with 1 of the patients also having hydronephrosis. Of the 4 patients, 2 were successfully managed by surgical removal of the old ileal conduit and creation of a new conduit; 1 required only maintenance of urinary drainage and recovered without any consequence; 1 with concurrent bilateral hydronephrosis was successfully treated by endoscopic ureteral stenting and maintenance of urinary drainage. Bilateral ureteroileal anastomosis obstruction with hydronephrosis was seen in 1 patient who also had hypoproteinemia. This patient recovered with temporary haemodialysis and nutritional support. One patient with unilateral ureteral dilation and hydronephrosis required unilateral nephrostomy. Another patient with unilateral ureteroileal anastomosis obstruction and hydronephrosis and normal renal function was managed with close surveillance only. In both patients, ureteroileal anastomosis obstruction and hydronephrosis disappeared within 6 months after surgery.

### Late complications related to the stoma and ureteroileal anastomosis

A total of 25 late complications related to the stoma and ureteroileal anastomosis were seen in 21 (21.0%) patients in the conventional ileal conduit group. No late complications occurred in the modified ileal conduit group.

Stoma-related late complications were seen in 13 patients 10–108 months after surgery; 5 patients had parastomal hernia alone, 2 had stomal prolapse alone, 1 had stomal retraction alone, 1 had stomal stenosis alone, 1 had both parastomal hernia and stomal prolapse, and 3 had both stomal retraction and stomal stenosis. Surgical intervention was required for 3 patients with severe parastomal hernia (including 1 with concurrent stomal prolapse) and all 3 patients with both stomal stenosis and stomal retraction.

Ureteroileal anastomosis obstruction with hydronephrosis was seen in 8 patients (unilateral in 6 patients and bilateral in 2 patients) 6–84 months after surgery. Two patients with bilateral obstruction at the ureteroileal anastomosis were successfully managed with open surgical repair. One patient who underwent unilateral nephrostomy for ureteroileal anastomosis obstruction was kept under close surveillance and recovered without any intervention. The remaining 5 patients had mild unilateral hydronephrosis and normal renal function; they were kept under close surveillance, and the hydronephrosis was resolved gradually.

Renal function was assessed by detecting serum creatinine levels. Preoperatively, renal function was normal in 208 patients; the remaining 37 had elevated creatinine level (> 132 μmol/L). After ileal conduit urinary diversion, renal function was deteriorated in 34 patients (20 in the modified ileal conduit group and 14 in the conventional ileal conduit group) at a median of 21 months (range, 0.2–84 months).

### Multivariable logistic regression and multivariable Cox regression analyses

For early and late complications, multivariable analyses revealed that patients in the modified ileal conduit group were significantly less likely to have stoma-related complications (odds ratio [OR] = 0.024, 95% confidence interval [CI] 0.003–0.235; *P* = 0.001) and ureteroileal anastomosis-related complications (OR = 0.141, 95% CI 0.042–0.476; *P* = 0.002). For early complications, multivariable logistic regression analyses revealed that patients in the modified ileal conduit group were significantly less likely to have stoma-related complications (OR = 0.211, 95% CI 0.027–0.689; *P* = 0.020) and ureteroileal anastomosis-related complications (OR = 0.314, 95% CI 0.077–0.831; *P* = 0.012) (Table 4). Because no late stoma-related and ureteroileal anastomosis-related complications occurred in the modified Bricker group, ileal conduit surgery was not included in multivariable analyses on late complications. Multivariable Cox regression analyses on other variables are shown in Table 5.

**Table 4:** Multivariable logistic regression analysis for predictors of early complications related to stoma and ureteroileal anastomosis in bladder cancer patients after ileal conduit surgery

Variable	Stoma-related complications		Ureteroileal anastomosis-related complications	
	OR (95% CI)	*P* value	OR (95% CI)	*P* value
Ileal conduit surgery (modified vs. conventional)	0.211 (0.027–0.689)	0.020	0.314 (0.077–0.831)	0.012
Age (> 62 vs. ≤ 62)	0.036 (0.001–2.866)	0.136	0.138 (0.008–3.499)	0.281
Gender (female vs. male)	5.949 (0.159–19.235)	0.334	2.003 (0.412–9.753)	0.390
Smoking (yes vs. no)	0.740 (0.185–7.682)	0.272	0.459 (0.114–1.847)	0.273
CCI (> 2 vs. ≤ 2)	2.643 (0.330–11.160)	0.173	3.541 (0.977–12.831)	0.054
ASA (> 2 vs. ≤ 2)	1.445 (0.152–8.473)	0.275	1.413 (0.292–6.852)	0.248
Prior pelvic irradiation (yes vs. no)	1.013 (0.183–6.592)	0.562	1.256 (0.089–7.697)	0.465
Preoperative hydronephrosis (yes vs. no)	1.211 (0.228–6.592)	0.352	2.069 (0.603–7.098)	0.248
Pathologic T stage				
T2 vs. T1	1.893 (0.564–8.259)	0.652	2.609 (0.376–10.119	0.332
T3 vs. T2	2.351 (0.268–9.543)	0.342	1.769 (0.669–8.629)	0.231
T4 vs. T3	1.367 (0.327–6.562)	0.103	1.749 (0.594–7.540)	0.308
Pathologic N stage (N + vs. N0)	1.534 (0.351–10.265)	0.065	1960 (0.219–13.560)	0.125
Adjuvant chemotherapy (yes vs. no)	1.125 (0.056–12.546)	0.218	1.683 (0.852–7.007)	0.172
BMI (> 21.8 vs. ≤ 21.8)	1.879 (0.253–6.753)	0.097	1.411 (0.406–4.906)	0.189
Year of surgery (> 2009 vs. ≤ 2009)	0.783 (0.073–2.126)	0.348	0.401 (0.097–1.652)	0.206

*OR* odds ratio, *CI* confidence interval, *CCI* Charlson comorbidity index, *ASA* American Society of Anesthesiology, *BMI* body mass index

**Table 5:** Multivariable Cox regression analysis for predictors of late complications related to stoma and ureteroileal anastomosis

Variable	Stoma-related complications		Ureteroileal anastomosis-related complications	
	HR (95% CI)	*P* value	HR (95% CI)	*P* value
Age (> 62 vs. ≤ 62)	0.238 (0.023–2.220)	0.094	0.042 (0.001–1.894)	0.103
Gender (female vs. male)	1.674 (0.401–8.994)	0.139	1.266 (0.108–4.569)	0.292
Smoking (yes vs. no)	0.958 (0.084–10.930)	0.973	0.055 (0.002–1.435)	0.081
CCI (> 2 vs. ≤ 2)	1.950 (0.330–11.509)	0.461	1.149 (0.111–11.855)	0.207
ASA (> 2 vs. ≤ 2)	1.852 (0.165–10.225)	0.203	1.289 (0.041–13.672)	0.185
Prior pelvic irradiation (yes vs. no)	1.938 (0.629–9.369)	0.292	2.325 (0.537–8.572)	0.420
Preoperative hydronephrosis (yes vs. no)	1.980 (0.188–10.980)	0.287	3.759 (0.470–14.456)	0.119
Pathologic T stage				
T2 vs. T1	2.259 (1.094–8.459)	0.043	2.875 (1.012–5.297)	0.032
T3 vs. T2	1.893 (0.154–8.349)	0.174	1.432 (0.023–11.242)	0.282
T4 vs. T3	1.564 (0.155–11.025)	0.131	1.973 (0.142–9.126)	0.259
Pathologic N stage (N + vs. N0)	2.252 (0.245–12.372)	0.219	3.123 (0.263–10.282)	0.075
Adjuvant chemotherapy (yes vs. no)	2.036 (0.242–6.734)	0.178	1.552 (0.018–6.723)	0.571
BMI (> 21.8 vs. ≤ 21.8)	2.594 (0.121–7.783)	0.067	1.934 (0.119–6.524)	0.053
Year of surgery (> 2009 vs. ≤ 2009)	2.125 (0.314–8.875)	0.257	2.538 (0.423–7.243)	0.689

*HR* Hazard ratio

## Discussion

This study aimed to compare the rates of early and late stoma- and ileal anastomosis-related complications between patients undergoing conventional and modified ileal conduit surgery for bladder cancer. The rates of both stoma- and ureteroileal anastomosis-related complications were significantly lower in the modified ileal conduit group than in the conventional ileal conduit group.

Ileal conduit diversion may lead to different types of complications, including stoma- and ureteroileal anastomosis-related problems, bowel obstruction, impaired renal function, infection, and so on [15]. Stoma- and ureteroileal anastomosis-related complications are the most common, comprising 25.0%–60.0% of all complications [16]. Stoma-related complications occur in 12.2%–42.3% of patients and ureteroileal anastomosis-related complications in 4.9%–14.0% of patients after ileal conduit diversion (Table 6) [10, 14, 15, 33–38]. A recent study found that parastomal hernia occured in 27.0% patients at 1 year and in 48.0% patients at 2 years after ileal conduit surgery [17]. In the present study, the rates of early and late stoma- and ureteroileal anastomosis-related complications after conventional ileal conduit urinary diversion was comparable to those in previous reports (Table 6) [10, 14, 15, 33–38]. However, after the modified ileal conduit surgery, the rates of early stoma- and ureteroileal anastomosis-related complications were only 0.7% and 4.8%, and no late stoma- and ureteroileal anastomosis-related complications occurred, suggesting that the modified ileal conduit technique was effective in reducing early complications and preventing late complications.

**Table 6:** Summary of literature reporting late complications related to stoma and ureteroileal anastomosis in bladder cancer patients after ileal conduit surgery

Reference	Total (cases)	Median/mean follow-up (months)	Complication [cases (%)]	
			Stoma-related	Ureteroileal anastomosis-related
Kouba et al. [10]	137	29	21 (15.3)	–
Madersbacher et al. [14]	131	98	31 (23.7)	18 (13.7)
Shimko et al. [15]	1057	186	163 (15.4)	106 (10.0)
Cheung et al. [33]	123	38	52 (42.3)	–
Gburek et al. [34]	66	20	–	6 (9.1)
Wood et al. [35]	93	63.4	32 (34.4)	–
Hétet et al. [36]	246	24	45 (18.3)	12 (4.9)
Jakko et al. [37]	118	> 12	14 (11.9)^a^	13 (11.0)
Movassaghi et al. [38]	92	34	21 (22.8)	–
The present study				
Conventional technique	100	32	13 (13.0)	8 (8.0)
Modified technique	145	30.5	0	0

Modified ileal conduit urinary diversion (the link to the video of surgical process: http://www.cancercommun.com/video/18-00281.asp)

– Not reported

^a^Parastomal hernia and stoma stenosis were included simultaneously

Our modified technique for creating ileal conduit is distinguished by three key characteristics that help reduce complications. First, a 15-cm distal ileal segment with adequate blood supply is isolated, and the stoma is created intracorporeally with excellent eversion. The skin at the stoma site is removed and the fascia is incised adequately to allow passage of two fingers; we believe that this contributes greatly to the prevention of stomal stenosis. Second, the ileal conduit is fixed to the abdominal wall fascia with 6 nonabsorbable sutures (instead of 4 absorbable sutures used in conventional ileal conduit surgery) and the stoma is sutured to the skin by interrupted sutures that incorporate the skin, the entire thickness of the edge of the bowel wall, and the underlying seromuscular layer; we believe that this measure helps prevent stomal retraction and prolapse. Third, the peritoneum underlying the stoma is left intact, and the whole ileal conduit is extra-peritonealized completely; this step helps prevent parastomal hernia.

Ureteroileal anastomosis-related complications are seen in 4.9%–14.0% of patients undergoing ileal conduit diversion [10, 14, 15, 33–38]. Ureteroileal anastomosis-related complications mainly result from deficiencies in the surgical technique. Li et al. [39] reported relatively few postoperative complications with their modified technique for ureteroileal anastomosis, in which the proximal end of the conduit is transferred to the left side of the sigmoid and the left ureter is anastomosed to the conduit in situ. In our modified technique, we incorporate two measures to reduce ureteroileal anastomosis-related complications. First, we use the technique of external sheath separation of the ureter to preserve its blood supply. Second, we ensure that the terminal ureter is spatulated adequately before it is anastomosed to the ileal conduit with a continuous lock-stitch suture. We believe that these modifications are responsible for the reduction in ureteroileal anastomosis-related complications.

The present study had a larger sample size and longer follow-up time than our previous study [23]. The results confirm our assumption that the modification of surgical technique to improve stoma creation and ureteroileal anastomosis can prevent or reduce complications related to stoma and ureteroileal anastomosis. However, this study has limitations. First, this is a retrospective study, and patients were not randomly assigned to undergo different surgical procedures; the selection of the surgical approach was mainly based on the surgeon’s experience and the patient’s preference. Therefore, patients in the two groups may not have been comparable in all respects, although the available baseline characteristics did not show any significant difference between the groups. Second, this study relied on clinical and radiological follow-up that was focused on the disease (bladder cancer) and not the stoma- and ureteroileal anastomosis-related complications. Therefore, it is likely that some stoma- and ureteroileal anastomosis-related complications may have been missed. Third, the majority of our patients had a normal BMI. High BMI is a known risk factor for parastomal hernia [10], and so our findings might not be entirely applicable to obese patients. A multicenter prospective randomized controlled clinical trial comparing stoma- and ureteroileal anastomosis-related complications between bladder cancer patients undergoing modified and conventional ileal conduit surgery is currently underway, and the findings should be able to clarify the benefits, if any, of the modified technique.

## Conclusions

Our modified ileal conduit technique appears to be effective for reducing and preventing early and late stoma- and ureteroileal anastomosis-related complications in bladder cancer patients undergoing ileal conduit urinary diversion. Prospective randomized controlled trials are needed to confirm our findings and the efficacy of this modified technique.

## Abbreviations

ASA: American Society of Anesthesiology
CDC: Clavien-Dindo Classification
OR: odds ratio
BMI: body mass index
IVP: intravenous pyelography
CT: computed tomography
ECOG: Eastern Cooperative Oncology Group
CCI: Charlson comorbidity index
IQR: interquartile ranges
SD: standard deviations
CI: confidence interval
HR: hazard ratio
